# Pulmonary and Chest Wall Metastasectomy in Urogenital Tumors: A Single Center Experience and Review of Literature

**DOI:** 10.5812/numonthly.17258

**Published:** 2014-05-03

**Authors:** Seyd Hossein Fattahi Masoum, Behzad Feizzdeh Kerigh, Alireza Goreifi

**Affiliations:** 1 Transplant Research Center, Imam Reza Hospital, Faculty of Medicine, Mashhad University of Medical Sciences, Mashhad, IR Iran; 2 Minimally Invasive Surgery Research Center, Kidney Transplantation Complications Research Center, Ghaem Medical Hospital, Faculty of Medicine, Mashhad University of Medical Sciences, Mashhad, IR Iran; 3 Department of Urology, Mashhad University of Medical Sciences, Mashhad, IR Iran

**Keywords:** Pulmonary, Thoracic Wall, urogenital Neoplasm, Neoplasm Metastasis

## Abstract

**Background:**

Pulmonary metastases are often found in advanced malignancies. Urogenital malignancies originating from kidney, prostate, testes, and bladder all metastasize preferentially to the lungs.

**Objectives:**

This retrospective study aimed to evaluate the results of pulmonary and chest wall metastasectomy in patients with primary urogenital Tumors.

**Patients and Methods:**

The patients who underwent pulmonary metastasectomy in Ghaem Hospital from 1996 to 2011 were examined. Thirteen out of 79 patients referred for pulmonary metastasectomy to a single thoracic surgeon had metastases from urogenital tumors; two cases with metastasis from urogenital tumors were inoperable. We reviewed their demographic data and also clinicopathological features. Disease free interval (DFI) was defined as the time between the first curative surgery and the appearance of the signs and symptoms of pulmonary metastasis.

**Results:**

Among 11 patients who underwent surgery consisted of eight males and three females. Their metastasis originated from testis tumors (n = 5), renal cell carcinoma (RCC; n = 4), bladder tumor (n = 1), and prostate cancer (n = 1). Their mean age was 41.27 years (range, 21-67). The mean age of the patients with RCC and testis tumor at the time of diagnosing metastasis was 54 and 24.8 years, respectively. There were two other patients (a 62-year-old female and a 54-year-old male) with pleural effusion due to metastatic RCC whose tumor was inoperable because of their poor general condition and hence, were referred for chemotherapy.

**Conclusions:**

Pulmonary metastasectomy is feasible in selected cases.

## 1. Background

Pulmonary metastases are often found in advanced malignancies. Urogenital malignancies originating from kidney, prostate, testicles, and bladder tumors metastasize preferentially to the lungs. The diagnosis of lung metastasis is often associated with a very poor prognosis and a short survival time. Consequently, few patients survive more than one year after diagnosis. In these situations, palliative chemotherapy is usually initiated; however, the possibility of metastasectomy should be considered (1, 2). Many studies have been conducted on pulmonary metastasectomy and it has become the standard of therapy for various lungs metastases from solid malignancies. Metastases of the primary tumors that do not respond well to chemotherapy, radiotherapy, or a combination of them are especially well suited for surgical resection. If metastases are restricted to the lungs, the use of surgery along with the overall oncological treatment is justified. In patients with widespread diffuse pulmonary metastasis or in those whose lesions are technically or functionally inoperable, local interventions such as surgery and radiotherapy are at best palliative. The standard procedure is a circumscribed atypical (lung tissue sparing) resection; rarely, anatomical resection such as pulmonary segmentectomy or lobectomy is required (1, 3).

## 2. Objectives

In this study, we presented our single center experience with pulmonary and chest wall metastasectomy of urogenital cancers and reviewed the studies concerning this issue.

## 3. Patients and Methods

We retrospectively examined patients who underwent pulmonary metastasectomy in Ghaem Hospital from 1996 to 2011. From 79 patients referred for pulmonary me tastasectomy to a single thoracic surgeon, there were 13 cases of urogenital metastases of which two cases were inoperable. We reviewed their demographic data as well as clinicopathological features. Disease free interval (DFI) was defined as the time between the first curative surgery and the appearance of signs and symptoms of pulmonary metastasis.

## 4. Results

Patients with metastases originating from urogenital cancers who underwent surgery consisted of eight males and three females. Their primary tumor was testis tumor (n = 5), RCC (n = 4), bladder tumor (n = 1), and prostate cancer (n = 1). Their mean age was 41.27 years (range, 21-67). The mean age of patients with RCC and testis tumor at the time of diagnosing the metastasis was 54 and 24.8 years, respectively. There were two other patients (A 62-year-old female and a 54-year-old male) with pleural effusion due to metastatic RCC who were found inoperable due to their poor general condition and were referred for chemotherapy.

Clinical presentation of the metastasis was dyspnea and cough in five patients and hemoptysis in one patient. Five patients were asymptomatic and their metastasis was diagnosed in their routine follow-up. Mean DFI was 3.73 years (range, 1-6) in all patients and 2.75 and four years in patients with RCC and testis tumor, respectively.

The site of metastasis was chest wall in two, right lung in four, and left lung in four patients. One patient had bilateral lung metastases. All surgeries were done by a single thoracic surgeon. Two patients with chest wall involvement underwent tumor resection. Chest wall was reconstructed using latissimus dorsi muscle and Mersilene mesh. The other types of surgical procedures included lobectomy (three cases), metastasectomy (three cases), segmentectomy (two cases), and pneumonectomy (one patient; Table 1).

No major complication occurred during surgery and in postoperation period. All patients were discharged with good condition. One patient (case 6 in Table 1) had hemophilia that was prepared for surgery with 100 IU of factor VIII and did not experience any complication in perioperative period.

**Table 1.** Summary of Patients Data^a^

Case	Age, y/Gender	DFI	Initial Pathology	Site of Metastasis/Type of Surgery
**1**	60/M	3	RCC	Chest wall/resection and reconstruction
**2**	56/F	3	RCC	Lt lung/sup lobectomy and wedge resection
**3**	45/F	6	RCC	Both lungs/metastasectomy
**4**	55/M	1	RCC	chest wall/resection and reconstruction
**5**	67/F	4	Bladder TCC	Lt lung (sup lobe)/lobectomy
**6**	47/M	4	Prostate sarcoma	Lt lung (sup lobe)/lobectomy
**7**	26/M	6	Seminoma	Rt lung/Rt lung pneumonectomy
**8**	32/M	4	Mixed GCT of testis	Lt lung/wedge resection and segmentectomy
**9**	22/M	5	Mixed GCT of testis	Rt lung/metastasectomy
**10**	23/M	3	Mixed GCT of testis	Rt lung/Inf and Mid segmentectomy
**11**	21/M	2	Mixed GCT of testis	Rt lung/metastasectomy

^a^ Abbreviation: M, male; F, female; RCC, renal cell carcinoma; TCC, transitional cell carcinoma (urothelial carcinoma); DFI, disease free interval; GCT, germ cell tumor; Rt, right; Lt, left; Sup, superior; Inf, inferior; and Mid, middle.

## 5. Discussion

Systemic metastases occur in about 20% of testicular germ cell tumors (GCTs), 25% to 30% of prostate cancers, 30% of urothelial carcinoma with muscle invasion, and 50% of RCCs; in addition, all of them metastasize preferentially to the lungs (4). We reviewed the studies concerning pulmonary metastasectomy in urogenital tumors as well as presenting our experience in this issue.

### 5.1. Renal Cell Carcinoma

RCC metastasizes preferentially to the lung either hematogenous or via lymphatic route (1). At the time of diagnosis, about 20% of patients show regional or systemic metastases and up to 30% of them present with metastases after radical nephrectomy (5). Lung and lymph nodes are the most frequent sites of metastasis (62%-77% and 34%-58%, respectively); the skeletal system and liver are involved less frequently (18%-30%) (4, 6).

Although development of new drugs like tyrosine kinase inhibitors and mTOR (mammalian target of rapamycin) inhibitors is associated with an improvement in progression-free survival, lung metastasectomy remains as an acceptable choice for curative treatment (7). In 21 studies published from 1961 to 2011, the five-year survival after RCC pulmonary metastasectomy ranged from 21% to 60% (8). on the other hand, in a study of eight selected patients without extrapulmonary metastases who underwent complete resection of solitary or multiple unilateral or bilateral RCC pulmonary metastases, five-year survival was 83% (9). In most studies, complete resection of metastasis was a good prognostic factor. Although resectability itself may be associated with better prognosis, reduction of tumor burden may also play a significant role. Higher number of metastases and lymph node involvement indicate advanced disease and may compromise complete resectability (8, 10). Other good prognostic factors include solitary metastasis (vs. multiple), smaller number of metastases (< 3 or < 7), smaller size of metastases (< 3 cm), metachronous (vs. synchronous) metastases, longer DFI (> 12-36 months), absence of positive hilar and/or mediastinal lymph nodes, absence of pleural infiltration, and absence of positive lymph nodes at initial nephrectomy (8). Simultaneous metastasis of thoracic lymph nodes occurs in 30% to 45% of cases and is associated with a much less favorable prognosis. Mean survival of these patients has been reported as 26 to 29 months, which is shorter in comparison to 64 to 92 months in patients without lymph node metastases. Therefore, patients with mediastinal lymph node metastases are not suitable for curative surgery (11). Surgery should resect all existing metastases, which may leads to a five-year survival rate of 40% to 50% and a mean survival time from 35 to 55 months, with low surgical mortality and morbidity rates (0%-2% and 1.5%-10%, respectively) (4). Pastorino et al. reported that the five- and ten-year survival rates of patients who underwent complete surgical resection were 36% and 26%, respectively, which were longer in comparison with the rates in those with incomplete resections (13% and 7% in five- and ten-years rates, respectively) (12). Tanguay et al. reported the potential benefits of combining surgery with systemic therapy. Median time to relapse was six months in patients treated with initial surgery and 8.5 months in patients treated with delayed surgery, although the latter group of patients had substantially more sever disease. Among the patients who underwent initial surgery, 55% had survived after median follow-up of 48 months while 66% of patients who received initial systemic therapy had survived 27 months. The authors emphasized that the disease burden was much greater in patients who had received initial systemic therapy; however, the promising results seem to support initial systemic therapy for patients with greater disease burden (13). Although DFI, defined as the time interval between nephrectomy and pulmonary metastasectomy or between nephrectomy and diagnosis of pulmonary metastases, varies in metachronous metastases, it does not exceed five years in the majority of cases. However, some others reported a maximum DFI of longer than 15 years (14, 15). Very late metastasectomy has been rarely reported, and the long-term results are practically lacking (8).

In our experience, we had four cases of metastatic RCC with mean DFI of 2.75 years, and the mean age of patients at the time of diagnosis of metastasis was 54 years. Two patients had chest wall involvement and two others had lung involvement. Moreover, we had two patients with pleural effusion due to metastatic RCC who were inoperable.

### 5.2. Testicular Tumors

The lung is the most common site of metastases in patients with testicular GCTs (16). Pulmonary metastasectomy in GCTs results in five-year survival rate of 65% (12), the best survival rate in comparison to the rates from metastasectomy of other tumor types including epithelial tumors, sarcomas, and melanomas. The role of pulmonary metastasectomy in the treatment of testicular GCT has been evolving with the introduction of cisplatin to chemotherapy regimens. Before cisplatin era, the five-year survival rate for patients after pulmonary metastasectomy was 41% that increased to 65% after introducing cisplatin (17).

Numerous studies have demonstrated the prognostic value of the resected residual lesions histology. In their study, Einhorn et al. concluded that surgical resection of residual disease following chemotherapy-induced cytoreduction with platinum combination chemotherapy may be therapeutic in some cases and helps to define the optimal subsequent treatment strategy (18). In nonseminomatous testicular cancer, all lesions that remain after chemotherapy should be removed because in up to 35% of cases, their histopathological findings are inconsistent with their retroperitoneal findings (1, 4). Normalization of the tumor markers after chemotherapy does not imply that removing the residual tumors in the lungs and mediastinum is not needed (1). Patients with elevated serum markers were traditionally believed as unresectable and not suitable for surgery; however, a few studies have demonstrated that salvage surgery may have potential for cure in a selected patients with lesions limited to one site (17). Murphy et al. reported that six patients with disease limited to the lung parenchyma and mediastinum had no evidence of disease with a minimum follow-up period of 31 months (19). Wood et al. reported that metastasectomy was performed for three patients with lung metastases and one with mediastinal disease of which two patients were disease-free at 16-month later follow-up (20). Moreover, in a study by Liu et al., eight patients had persistently elevated serum tumor markers after chemotherapy prior to pulmonary metastasectomy. After metastasectomy, five patients survived without evidence of disease, three of which were considered as cured with follow-up period of longer than 15 years (17).

In general, indications of resecting lung metastatic foci include residual tumors after chemotherapy and normalization of tumor marker values, lack of response to chemotherapy, partial response to chemotherapy, and recurrence after chemotherapy (1). The histological discordance between the two lungs is only 5%; hence, the decision for or against surgical resection of contralateral metastasis may be made on the basis of histological findings of the first procedure (4).

In our study we had five cases of testicular tumor, four cases of mixed GCTs and one case of seminoma. All of them had normal tumor markers before metastasectomy and their mean DFI was four years.

### 5.3. Urothelial Carcinoma

Locoregional or systemic recurrence may be seen respectively in 5% and 35% of patients with muscle-invasive urothelial carcinoma (transitional cell carcinoma) of the urinary bladder after radical cystectomy (21, 22). The standard treatment consists of systemic chemotherapy which leads to partial or complete remission in 70% of patients with mean progression-free interval of seven months, and shorter than 14 months mean survival time (23). Recurrences occur usually at the site of the primary metastasis, which indicates persistence of active cancerous cells; hence, metastasectomy is reasonable to improve the prognosis (4).

Cowles et al. reported surgery for metastatic urothelial cancer for the first time. They observed a long-term disease control in six patients after the resection of a solitary pulmonary metastasis (24). Siefker-Radtke et al. reported outcomes of 31 patients undergoing postchemotherapy resection of metastases of which 77% were lung metastases. Overall survival (OS) since the time of surgery was 23 months and five-year survival rate was 33% (25). Abe et al. observed a median survival time of 42 months in 12 patients, with visible cancer in 83% of them, who underwent metastasectomy at multiple sites including the lungs (26). In a recent study by Lehmann et al., outcomes of 44 patients from 15 different centers in Germany were reported. The patients had distant metastases of the urothelial or upper urinary tract tumors and underwent complete resection of all detectable metastases. OS from the time of resection was 27 months and 7 (15.9%) patients survived for more than two years without disease progression (27). Recently, Siefker-Radtke et al. presented data from a phase II clinical trial of sequential neoadjuvant chemotherapy with ifosfamide, doxorubicin, and gemcitabine followed by cisplatin, gemcitabine, and ifosfamide in locally advanced urothelial carcinoma. In their report, 35 patients underwent surgical consolidation, including 24 with nodal metastasis, six with tumor fixed to the pelvic sidewall, and five with metastasis to other organs such as lung, brain, abdominal wall, or ileum. Five-year OS for these patients was 29%; moreover, the greatest improvement was seen in patients undergoing surgical consolidation after a 90% or greater response to chemotherapy (28).

The available evidence suggests that a selected group of patients benefit from surgical consolidation of visceral metastases, most frequently in the setting of lung metastasis. Although there is no standard guideline in this setting, Svatek et al. considered surgical consolidation of visceral metastases for patients with tumor at one distant site who had responded well to chemotherapy and had no evidence of rapid progression elsewhere. In addition, before considering surgical consolidation, they observed patients for three to six months following chemotherapy to exclude rapid progression (29).

In our experience, we had only one case of bladder carcinoma that underwent pulmonary lobectomy with DFI of four years.

### 5.4. Prostate Carcinoma

Pulmonary metastases are found in 5% to 27% of prostate cancer cases and usually only after bone metastasis (30, 31). These metastases may present in a diffuse interstitial pattern representing lymphatic spread, which is the most common form, or a multinodular pattern representing hematogenous spread, which is seen in 8% to 20% of those with positive radiographic findings. Solitary pulmonary nodules have been reported but are extremely rare (32). Recently, modern diagnostic imaging methods such as choline PET/CT (positron emission tomography/computed tomography) have become essential tools by enabling early diagnosis of isolated metastases in patients with low-PSA recurrence, which can raises patients’ hopes of cure (33). Salvage metastasectomy is a surgical treatment of locoregional or solitary systemic metastases that are confirmed by imaging studies after local primary or systemic therapy. The aim of salvage metastasectomy in prostate cancer is to gain time before initiating systemic androgen deprivation, which is associated with marked side effects that are time-limited. This treatment is not helpful to increase the survival time. Due to the limited life expectancy, visceral metastasectomy or, alternatively, radiotherapy should be performed only if the patient’s symptoms cannot be controlled by conservative management or if his function is threatened (4).

In our experience we had only one case of prostate sarcoma which underwent lobectomy with DFI of four years. The patient has been operated because of his severe respiratory symptoms that could not be controlled by conservative management.

The diagnosis of lung metastases in urogenital tumors is often associated with a very poor prognosis and a short survival time. The primary treatment in these patients is palliative chemotherapy. However, an interdisciplinary tumor board should also discuss the possibility of metastasectomy. The aim of treatment in these situations is palliative local radical resection and symptom control. Survival after lung metastasectomy depends on the nature of the primary tumor. In the case of urogenital tumors, pulmonary metastasectomy can be performed in selected patients and may improve the survival rate as well as quality of life. Therefore, the decision for or against metastasectomy must be made on a case-by-case basis.
